# The Relationship between Metabolic Control and Growth in Children with Type I Diabetes Mellitus in Southwest of Iran

**DOI:** 10.1155/2015/917542

**Published:** 2015-09-20

**Authors:** Shide Assar, Koroush Riahi, Shiva Bashirnezhad, Leila Yazdanpanah, Seyed Mahmoud Latifi

**Affiliations:** Health Research Institute, Diabetes Research Center, Ahvaz Jundishapur University of Medical Sciences, Ahvaz, Iran

## Abstract

*Background*. Metabolic control is an important factor in growth of children with type I diabetes. This study assessed the relationship between growth and metabolic control in such children. *Materials and Methods*. 83 children with diabetes were studied. They were examined for weight and height gain and HbA1C was quantified every 3 months for one year. The growth process was studied in patients who were divided into 3 groups according to their HbA1C amounts, consisting of good, intermediate, and poor metabolic control. *Results*. Mean age of cases was 7.6 ± 2. The presenting sign at the onset of disease was diabetic ketoacidosis in 44.6%. The average HbA1C amount was 8.89%. The average weight SDS at diagnosis was −0.18 and at the end of the study was 0.45 (*P*<0.001). The average height SDS at diagnosis was −0.04 and at the end of the study was −0.07 (*P*=0.64). A significant difference in weight SDS changes was only seen between patients with good and poor metabolic control (*P*=0.04). *Conclusion*. Poor metabolic control can decrease height growth but has minimal influence on weight. Metabolic control was not the only predictive factor of physical growth in children with diabetes.

## 1. Introduction

Diabetes is one of the main problems in health systems in the world [1]. Diabetes mellitus (DM) is a common chronic metabolic disorder and a main problem in health systems [2] with two main types: type I, in which the secretion of insulin from the beta cells is reduced or absent, and type II, which shows decreased insulin or insulin resistance in skeletal muscle, liver, and adipose tissue [3–6]. The onset of type I DM is usually during childhood, at an average age of 7 to 15 years; however this disease can be seen at any age [7, 8]. It is an autoimmune disease that is correlated with both genetic and environmental factors [9, 10]. Children with diabetes may experience delayed puberty and eventually reach a lower height than their genetic potential. Normal growth is one of the goals in the treatment of children with diabetes. The use of insulin regimens in patients with newly diagnosed insulin-dependent DM has substantially improved final height prognosis. Insulin treatment may prevent disorders of the growth hormone axis and allow normal growth in children with diabetes [11–13]. However, some studies have shown that the growth rate in such children is normal and is not related to HbA1C levels. These studies have concluded that the main determinant of growth is not metabolic control because moderate metabolic control can also lead to normal growth [14]. Despite the fact that type I diabetes, like other chronic diseases, may have negative effects on growth, it is not clear if the growth is only affected by the duration and control of DM or if other contributing factors also exist [15, 16]. According to some studies, patients with type I DM beginning at ages under 5 years have the highest rates of deficiency in height growth during puberty [17, 18]. Also a relationship between the onset of DM at a lower age and growth deficit has been demonstrated [19–21]. However others have reported that children with diabetes were shorter in height at diagnosis compared to healthy children [22, 23], in which the age of diagnosis has apparently been the most important factor affecting the results of these studies [20]. Given the prevalence of DM and the importance of growth in children, different results were reported in different studies on growth and related factors in children with diabetes. The aim of this study was to determine the relationship of metabolic control and growth in children with DM referred to Golestan Hospital diabetes clinic in Ahvaz, southwest of Iran, and the prevalence of growth retardation in such children.

## 2. Method and Material

Children with diabetes who were referred to diabetic clinic in Ahvaz Golestan Hospital from 2010 were included in the study. A written consent was obtained from the participants' parents. This study was approved by ethical committee of Ahvaz Jundishapur University of Medical Sciences. Inclusion criteria were age of 1–15 years and signing the informed consent by their parents. Exclusion criteria were hypothyroidism, celiac disease, thalassemia, and other diseases that have effect on growth. Age, height, and weight at the time of diagnosis and follow-up visits were recorded. Events of ketoacidosis were recorded. Patients were followed every 3 months. A detailed history and complete physical examination were performed. Exact measurements of height and weight, number of admissions with ketoacidosis, type of insulin, and its dosage were also recorded. Standing height without shoes (with Seca height meter) and weight without jackets and blazers were measured with standard scales, in the clinic. NCHS percentile for height and weight was determined based on standard curves. Based on height and weight, BMI and height SDS were calculated according to the following formulas:

1. BMI = (weight in kilograms/square of height in meters).
2. Height SDS = [2 × (50 height percentile − patients height)/(50 percentile height − 3 percentile height)].

In this study HbA1C was measured in laboratory of diabetes research center of Ahvaz Jundishapur University of Medical Sciences. Values between 6 and 7.9% were considered as good metabolic control, values within 8–9.9% were considered as intermediate, and values of 10% or higher were considered as poor metabolic control. Seventeen patients were excluded from our study (12 patients due to lack of regular follow-up, 2 patients for thyroid disease, one patient due to celiac disease, and two patients because of migration) and 83 patients were finally included and each patient was followed for one year. The patients used three types of insulin therapy. In the first method, fixed doses of NPH and regular insulin were used in the morning and evening. In the second method a fixed dose of NPH insulin was used in the morning and evening and doses of regular insulin were adjusted according to blood glucose levels obtained by glucometers and injected three times a day (morning, noon, and before dinner). In the third method, long-acting insulin was used at bedtime and short-acting insulin was administered before meals. Data analysis was performed by chi-square, repeated measure, ANOVA, and Pearson's correlation coefficient tests. *P* value less than 0.05 was considered as significant.

## 3. Findings

**Figure 1:**
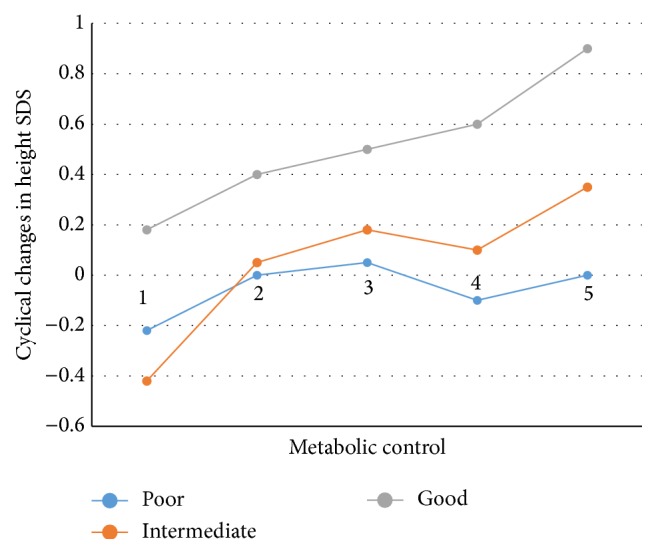
The mean of height SDS of patient separated metabolic control.

In this study, 83 patients (53% females and 47% males) were studied. Mean age of cases was 7.6 ± 2.9 (range: 2.5–13.5). Duration of DM was < 1 year in 61.4% and > 1 year in 38.6% of cases. Diabetic ketoacidosis was the diagnostic key in 44.6% of subjects (52.3% of girls and 35.9% of boys). Diabetic ketoacidosis was seen one time in 14.5% and repeated two times in 1.2% of subjects; however 70% of the participants did not experience diabetic ketoacidosis during this study. Age range of menarche was 11–13.83 years. The average age of onset of menarche in girls was 12.8 years. Nine patients had experienced menarche before the study. Of all patients 54.4% have used a fixed dose of NPH and regular insulin twice a day, 21.7% have used the second method, and 22.9% used the third method of insulin therapy. Gender and age groups did not significantly influence the types of insulin regimens. The mean HbA1c level in patients was 8.89, ranging from 5.85 to 14.25. The mean HbA1c was 9.07 in girls and 8.70 in boys. Average HbA1c level at ages younger than 5 years was 8.67, for ages 5 to 10 years was 8.70, and in ages 10 years and older was 9.50. Differences in mean HbA1c levels based on age and sex were not statistically significant (*P* = 0.21 and 0.3, resp.). Based on metabolic control 20.5% of patients were in the poor metabolic control group (HbA1c: 10% or higher). 42.2% of subjects were placed in moderate metabolic control group (HbA1c: 8–9.9%) and 37.3% were placed in good metabolic control group (HbA1c: 6–7.9%). No significant difference was observed between the metabolic control and sex and age (*P* = 0.29 and 0.58, resp.) (Table 1). To determine the growth status of children with DM at diagnosis, height percentile, weight, height and weight SDS, and BMI were used. None of the patients were below the third percentile for height at diagnosis. 1.2% of patients had a weight below the third percentile. In terms of BMI, 9.6% of patients were less than the fifth percentile. Changes in mean BMI were seen in all patients during the study. This increase was significant in both genders and all three age groups (*P* < 0.001). Average weight SDS at diagnosis was −0.18 and at the end of the study was 0.45 (*P* < 0.001). The increase of weight SDS was significant in both gender groups and in all patients under 10 years old. Average height SDS at diagnosis was −0.04 and at the end of the study was −0.07 (*P* = 0.64). The decrease in height SDS in both genders and in all age groups was not significant. When the average weight SDS of each group was compared with the two other groups of metabolic control (one-way ANOVA and Tukey's test) a significant increase was seen between average weight SDS of patients with good metabolic control compared to patients with poor metabolic control (*P* = 0.04) (Table 2). A significant increase was seen in comparing the changes in height SDS and their metabolic control by ANOVA (*P* = 0.017). This significant increase was also seen in the analysis of comparison between poor-intermediate and poor-good metabolic control groups by Tukey's test (*P* < 0.04) (Table 3) (Figure 1). To determine the relationship between gender and metabolic control the chi-square test was used and significant differences were not observed (*P* = 0.29) (Table 1). There was no significant relationship between age and metabolic control using Fischer exact test (*P* = 0.58). The frequency of menarche based on metabolic control in this study was assessed using the Kruskal-Wallis test which was not significant (*P* = 0.96). A significant relationship was found between reduced height SDS and repeated ketoacidosis by *t*-test (*P* = 0.03). A significant relationship was not found between changes in height SDS, disease duration, and type of insulin therapy.

## 4. Discussion

The aim of this study was to determine the relationship of metabolic control and growth in children with DM. In this study 83 patients were studied. The mean age at onset of menarche in girls was 12.8 years which is two months later than mean age of Iranian girls (12.6) [24, 25]. Salardi and colleagues have reported that the mean age of menarche in diabetic girls was 12.5 years which is about 6 months later than the average for their age [11, 13]. Elamin and colleagues in Sudan also reported a delayed onset of menarche [16]. However, an obvious delay in the age of menarche was not reported by Donaghue et al. [26]. The mean HbA1c level in patients was 8.89%, ranging from 5.85 to 14.25%. Differences in mean HbA1c levels based on age and sex were not statistically significant. In Salardi et al.'s study the mean HbA1c in boys was 11.17% and was 11.96% in girls which was, respectively, higher than our patients [13]. Most participants were in the moderate metabolic control group (42.2%) and no significant difference was observed between metabolic control and sex and age, although children under 10 years have better metabolic control. Salardi et al. showed that in both gender groups children with diabetes were taller than children without diabetes at diagnosis [13]. However in our study only 31.8% of female patients and 35.9% of male patients had a height of 50th percentile for their ages at diagnosis but none of the patient had a height below the 3rd percentile. Average weight SDS at the onset of DM in girls was −0.44 and in boys was 0.10. Average height SDS at the onset of DM in girls was −0.16 and in boys was −0.08. It can be concluded from the above results that, at diagnosis, boys have better growth indexes than girls. The mean initial BMI was 16.13 and the final average BMI was 17.94; a significant increase in BMI was seen (*P* < 0.001) during the study. Changes in mean height SDS between the onset and end of the study indicate that height SDS was higher at the onset compared to height SDS after treatment which can be seen in both genders and in all ages. Such an outcome was also obtained by Salardi et al. [13]. Another study reported that at the time of diagnosis these children were taller than normal, but this was reversed during course of disease [27]. In this study, there was no significant difference between good metabolic control group and intermediate group of height SDS which means normal relative growth in diabetic children with an intermediate control can be expected. There was a statistical significant relationship between ketoacidosis and height growth (*P* = 0.04) and a decrease in height SDS was noticeable in patients who had endured ketoacidosis. Many studies have found similar results to our study [16, 26–31]. However in some studies [1, 3, 4] an association between metabolic control and height SDS values was not found. More boys were in the good metabolic control group than girls clinically (46.2% versus 29.5%) but the difference was not statistically significant. Though the relationship between age groups and metabolic control was not significant (*P* = 0.58), clinically, good metabolic control had a higher prevalence in the 5–10-year age group and poor metabolic control was seen the most in the 10-year and older age group. In this study there was no significant relationship method of insulin therapy and height SDS scores. Donaghue and colleagues demonstrated a relationship between less frequent insulin injections and a reduction in height SDS [26]. Rudolf and Jackson and colleagues showed that patients with high levels of metabolic control and intensive insulin therapy had a normal growth rate [32, 33]. In this study no significant correlation was found between growth and disease duration. Accordingly, patients were divided into two groups of patients: duration of DM less than one year (51 cases) and duration of DM more than one year (32 cases).There were no significant differences between these two groups in our study; but in another study height growth disorder had relationship with disease duration [34].

## 5. Conclusions

According to this study the only determinant of growth is not metabolic control; however poor metabolic control may reduce growth. Weight is less affected by metabolic control in comparison to height growth.

## Figures and Tables

**Table 1:** Comparison of relationship between gender and metabolic control.

Gender (sex)	Metabolic control	Poor	Intermediate	Good
Female	Number	10	21	13
	Percentage	22.7	47.7	29.5

Male	Number	7	14	18
	Percentage	17.9	35.9	46.2

Total	Number	17	35	31
	Percentage	20.5	42.2	37.3

We use chi-square test to determine the relationship between gender and metabolic control that there was no significant difference (*P* = 0.29).

Although this difference was not statistically significant, good metabolic control in male was higher than female (resp., 46.2% versus 29.5%).

**Table 2:** The comparison mean of weight SDS of patient separated metabolic control during study.

Metabolic control		Mean	Standard deviation (SD)	Sig.
Poor	Intermediate	−0.25	0.18	0.36
	Good	−0.46	0.18	0.04

Intermediate	Poor	0.25	0.18	0.36
	Good	−0.21	0.15	0.35

Good	Poor	0.46	0.18	0.04
	Intermediate	0.21	0.15	0.35

One-way ANOVA and Tukey's test were significant.

Relationship between poor and good group was significant (*P* = 0.04).

**Table 3:** The comparison mean of height SDS of patient separated metabolic control during study.

Metabolic control		Mean	Standard deviation (SD)	Sig.
Poor	Intermediate	−0.23	0.08	0.018
	Good	−0.21	0.08	0.038

Intermediate	Poor	0.23	0.08	0.018
	Good	0.02	0.07	0.95

Good	Poor	0.21	0.08	0.038
	Intermediate	−0.02	0.07	0.95

One-way ANOVA and Tukey's test were significant.

Relationship between poor-intermediate and poor-good group was significant (*P* < 0.04).
